# Systematic Comparison of 10X Genomics and Parse Single Cell RNA Technologies across PBMC and CD8 ^+^ TEMRA Cells

**DOI:** 10.64898/2026.09.02.748788

**Published:** 2026-09-06

**Authors:** Alejandro Espinoza, Bruno J. de Andrade Silva, Matteo Pellegrini

## Abstract

Single-cell RNA sequencing technologies provide insights into gene expression at the cellular level, enabling detailed analysis of cellular heterogeneity. In this study, we systematically compared two scRNA-seq platforms,10X Genomics and Parse Biosciences, using human peripheral blood mononuclear cells (PBMCs) and terminally differentiated effector memory CD8 ^+^ T cells (TEMRAs). We identified significant differences in gene expression variability and platform-specific biases, such as ribosomal and mitochondrial gene capture. 10X has a bias for shorter genes, while Parse exhibited enhanced detection of longer transcripts. In CD8 ^+^ TEMRAs, the expression of key genes related to antimicrobial immune responses were underrepresented in Parse cells compared to 10X cells (e.g. *GNLY* , *PRF1* and *GZMB* ). These findings underscore the need for careful selection of scRNA-seq platforms based on specific research objectives, as platform-specific biases can influence cell type identification and as well as mechanistic insights derived from gene expression data. Our results provide insights for selection of scRNA-seq experimental platforms in immunological studies.

## Full Text Availability

The license terms selected by the author(s) for this preprint version do not permit archiving in PMC. The full text is available from the preprint server.

